# Association of monocyte-lymphocyte ratio with peripheral arterial disease in US participants: a retrospective cross-sectional study

**DOI:** 10.3389/fcvm.2025.1613138

**Published:** 2026-01-08

**Authors:** Qiang Liu, Xing Wu, Jianjun Shi

**Affiliations:** 1 Department of Cardiovascular Surgery, Taiyuan Central Hospital, Taiyuan, China; 2 Department of Cardiovascular Surgery, The Ninth Clinical College Affiliated to Shanxi Medical University, Taiyuan, China; 3 Department of Nephrology, Taiyuan Central Hospital, Taiyuan, China; 4 Department of Nephrology, The Ninth Clinical College Affiliated to Shanxi Medical University, Taiyuan, China

**Keywords:** ankle-brachial index, cross-sectional study, monocyte-lymphocyte ratio, National Health and Nutrition Examination Survey, peripheral arterial disease

## Abstract

**Background:**

Peripheral arterial disease (PAD), a prevalent manifestation of atherosclerosis, is closely linked to systemic inflammation. The monocyte-lymphocyte ratio (MLR), a cost-effective inflammatory biomarker, has shown prognostic value in cardiovascular diseases but remains underexplored in PAD.

**Objective:**

This study aimed to investigate the correlation between MLR and the occurrence of PAD.

**Methods:**

Utilizing data from the National Health and Nutrition Examination Survey (NHANES) 1999–2004, this retrospective cross-sectional study applied complex survey weighting to generate nationally representative estimates. A total of 2,827 participants aged ≥40 years were included after applying sample exclusions and accounting for missing data. PAD was defined as an ankle-brachial index <0.9. The MLR was calculated from complete blood count data. Weighted multivariable logistic regression models were employed to examine the association between MLR and PAD, with sequential adjustment for sociodemographic, lifestyle, metabolic, and clinical covariates. Weighted restricted cubic spline regression was used to evaluate potential nonlinearity, and a two-piecewise logistic model was applied to identify inflection points through maximum likelihood estimation.

**Results:**

In the weighted sample, the prevalence of PAD was 3.96%. Participants in the highest MLR tertile were older, more often male, and had higher cardiovascular risk profiles. In unadjusted analyses, elevated MLR was strongly associated with PAD (OR = 6.52, 95% CI: 2.60–16.33). However, this association was substantially attenuated after full adjustment for covariates including age (OR = 1.99, 95% CI: 0.78–5.07). Nonlinear analysis revealed a significant threshold effect at an MLR of 0.343 (*P* for log-likelihood ratio test = 0.009). Below this inflection point, each unit increase in MLR was associated with a 62% higher odds of PAD (OR = 1.62, 95% CI: 1.24–2.11), whereas no significant association was observed above the threshold (OR = 0.94, 95% CI: 0.64–1.38). Subgroup analyses demonstrated consistent associations across demographic and clinical strata, with no significant interaction effects.

**Conclusions:**

This nationally representative analysis identifies a threshold-based association between MLR and PAD. Despite substantial confounding by age, elevated MLR—particularly in the highest tertile—remains an independent indicator of increased PAD risk, supporting its potential utility in clinical risk assessment.

## 1 Introduction

Peripheral arterial disease (PAD) is a form of atherosclerosis marked by the accumulation of fatty deposits within the arteries of the lower limbs and feet (1). It affects more than 200 million people worldwide, including over 8.5 million adults aged 40 and older in the United States, with similar prevalence rates observed in both men and women (2, 3). The economic and health impacts of PAD are considerable, underscoring the importance of identifying its underlying causes and implementing effective interventions. Previous studies have emphasized the critical roles of inflammatory markers, endothelial dysfunction, and oxidative stress in the pathogenesis of PAD (4–9).

Emerging evidence underscores chronic inflammation as a pivotal factor driving the progression of atherosclerosis in PAD (10). Monocytes are integral to vascular inflammation, as they infiltrate arterial walls and differentiate into lipid-laden macrophages. Simultaneously, lymphopenia serves as a marker of systemic inflammatory burden and immune dysregulation (11, 12). The monocyte-lymphocyte ratio (MLR), which reflects the interaction between these two counter-regulatory pathways, has gained recognition as a cost-effective inflammatory biomarker. A summary of the current evidence linking MLR to various cardiovascular conditions is provided in Supplementary Table S1. Recent studies have validated its prognostic significance in conditions such as coronary artery disease (13–15) and ischemic stroke (16, 17), yet its relevance to PAD remains inadequately explored. This gap in knowledge is particularly noteworthy given the distinct pathophysiological differences between peripheral and coronary atherosclerosis (18). Therefore, this study aims to investigate the clinical and predictive significance of MLR specifically among U.S. adults diagnosed with PAD.

Most existing research on hematologic indices in PAD has concentrated on the neutrophil-lymphocyte ratio (NLR) and the platelet-lymphocyte ratio (PLR) (19, 20). While these markers exhibit moderate predictive value, their clinical utility is often constrained by their sensitivity to acute phase responses and the effects of medications (21). In contrast, MLR may provide advantages by capturing both innate immune activation and adaptive immune exhaustion, potentially yielding a more stable inflammatory profile (22). Therefore, this study aims to investigate the clinical and predictive significance of MLR among U.S. adults diagnosed with PAD.

## 2 Materials and methods

### 2.1 Study population

In this retrospective cross-sectional study, we examined data from 2,827 participants in the National Health and Nutrition Examination Survey (NHANES) conducted in the United States from 1999 to 2004. NHANES collects a variety of health-related information, including demographic details, physical examination results, and laboratory findings (23). The data collection was approved by the ethics review board of the National Center for Health Statistics, with all participants providing written informed consent before their inclusion (24). The NHANES data can be accessed publicly through their website (http://www.cdc.gov/nchs/nhanes.htm).

The NHANES database is highly suitable for investigating PAD, as it provides standardized ankle-brachial index (ABI) measurements—a validated and widely accepted diagnostic criterion for PAD in epidemiological studies—alongside a comprehensive set of demographic, examination, and laboratory variables, enabling robust adjustment for potential confounders.

Of the initial 31,126 participants, we first excluded those younger than 40 years (*n* = 21,156) because ABI measurements were not performed in this age group. Further exclusions included participants with missing bilateral ABI data (*n* = 2,408), ABI values >1.4 (*n* = 65) (25, 26), or missing monocyte/lymphocyte counts (*n* = 248). After also removing individuals with incomplete data on covariates (education, marital status, income, physical activity, smoking, BMI, total cholesterol, CRP, and HbA1c), 6,118 participants were retained for the primary analysis. To improve national representativeness, we further excluded those with missing fasting weight data (*n* = 3,291), resulting in a final weighted sample of 2,827 participants.

### 2.2 Peripheral artery disease

ABI measurements were conducted on participants aged 40 years and older, following a specific methodology established in previous studies. After a brief rest period, systolic blood pressure readings were obtained from the right arm and both ankles. In cases where right arm measurements were unavailable, the left arm was utilized instead. Participants aged 40–59 had their systolic blood pressure measured twice, whereas those aged 60 and above were assessed once. The ABI was calculated by dividing the average systolic blood pressure of the ankle by that of the arm.

PAD was defined as an ABI of less than 0.9 in either leg, consistent with established epidemiological criteria (23, 27). The NHANES examination did not include assessments for upper limb artery disease. To account for medial arterial calcification—a common source of artifact in individuals with diabetes that causes incompressible arteries and falsely elevated ABI readings—we excluded participants with an ABI value greater than 1.4 in either leg (23). This standard practice enhances the diagnostic specificity of a low ABI for obstructive PAD within the study sample.

### 2.3 Study variables and outcome

MLR is calculated by dividing the monocyte count by the lymphocyte count, with both values readily available from laboratory data files.

We employed standardized surveys to collect socio-demographic and lifestyle data. The Mobile Examination Center provided examination outcomes, which included measurements such as body mass index (BMI), blood pressure, and various biochemical markers. Based on existing literature (27, 28–36), we examined several potential factors, including age, gender, ethnicity, education level, marital status, family income, smoking habits, physical activity levels, BMI, and laboratory test results (such as total cholesterol, C-reactive protein, and glycated hemoglobin A1c). Additionally, we considered pre-existing medical conditions, including diabetes, hypertension, and cardiovascular disease (CVD).

For more detailed information on these variables, including the measurement methods used in the questionnaires and a complete list of variables, please refer to the official NHANES website (https://www.cdc.gov/nchs/nhanes/). Family income was categorized into three groups based on the poverty income ratio (PIR): low (PIR ≤ 1.3), medium (PIR > 1.3–3.5), and high (PIR > 3.5). Smoking status was categorized according to definitions from previous research: never smoked, current smoker, and former smoker. To assess physical activity levels, participants reported on vigorous exercises that significantly increased breathing and heart rate (e.g., swimming or fast cycling) and moderate activities that caused mild to moderate physiological responses (e.g., golfing or leisurely biking). Each activity was required to have lasted at least 10 min in the past month. Physical activity levels were classified into three categories: below moderate (no moderate or vigorous activities), moderate (no vigorous exercise but at least one moderate activity), and high intensity (at least one instance of vigorous exercise).

Hypertension was defined as having an average systolic blood pressure (ASBP) or average diastolic blood pressure (ADBP) of 140/90 mmHg or higher, currently using antihypertensive medication, receiving a diagnosis from a healthcare provider, or meeting any combination of these criteria. Diabetes was characterized by a fasting glucose level exceeding 7 mmol/L, a random glucose level of 11.1 mmol/L or more, a glycated hemoglobin A1c of 6.5% or higher, the use of glucose-lowering medications, or a documented history of diabetes. The presence of cardiovascular diseases was assessed based on self-reported experiences with conditions such as congestive heart failure, coronary heart disease, angina pectoris, myocardial infarction, and stroke. BMI was calculated using the standard formula that involves an individual's weight and height.

### 2.4 Statistical analysis

In alignment with NHANES analytical recommendations, this secondary analysis accounted for the complex survey design by integrating sampling weights, stratification, and clustering variables to generate nationally representative estimates and minimize Type I error. Data from household interviews and Mobile Examination Center (MEC) examinations were combined, with fasting subsample weights (WTSAF2YR) applied to adjust for differential probabilities of selection and survey nonresponse. The survey design was implemented using the svydesign function in R, incorporating stratification (SDMVSTRA), primary sampling units (SDMVPSU), and appropriate weight adjustments to reflect the U.S. non-institutionalized population aged 40 years and older.

Categorical variables are summarized as unweighted counts with weighted proportions, and continuous variables as means with standard errors. Between-group comparisons employed weighted Student's *t*-tests for normally distributed continuous data, Mann–Whitney *U* tests for non-normally distributed variables, and chi-square tests for categorical variables. Covariates were selected based on clinical plausibility, univariate significance, and a pre-specified >10% change-in-estimate criterion for the MLR-PAD association. Variables meeting any of these criteria were included in final models. Supplementary Table S2 provides a complete assessment of each covariate's confounding influence and multicollinearity (VIF < 2.0), validating the approach.The relationship between MLR and PAD was evaluated through weighted multivariable logistic regression using a hierarchical adjustment strategy across five sequential models: Model 1 (unadjusted); Model 2 (adjusted for gender, race, education, marital status, poverty-income ratio, and physical activity); Model 3 (Model 2 + BMI, total cholesterol, CRP, HbA1c, and smoking status); Model 4 (Model 3 + history of cardiovascular disease, hypertension, and diabetes); Model 5 (Model 4 + age).

To examine potential nonlinearity, weighted restricted cubic spline (RCS) regression with four knots was fitted. If the nonlinearity test was significant (*P* < 0.05), a threshold-effect analysis was conducted using a two-piecewise logistic model, with the inflection point determined by maximum likelihood estimation. The log-likelihood ratio test compared the nonlinear model with a linear specification.

Subgroup analyses were performed to assess effect modification by age (<65 vs. ≥65 years), sex, physical activity level, family income, education, and comorbidities (cardiovascular disease, hypertension, diabetes). Heterogeneity across subgroups was evaluated with multivariate logistic regression, and interaction effects were tested via likelihood ratio tests.

Statistical analyses were performed using R Statistical Software (Version 4.2.2, http://www.R-project.org, The R Foundation) and the Free Statistics Analysis Platform (Version 2.1, Beijing, China, http://www.clinicalscientists.cn/freestatistics). A detailed explanation of their functionalities ensured transparency and reproducibility of the analytical methods used in this study. Overall, the statistical analysis appears to be comprehensive, well executed, and supported by appropriate software tools, contributing to the strength of the study's findings.

## 3 Results

### 3.1 Characteristics of the study population

**Figure 1:**
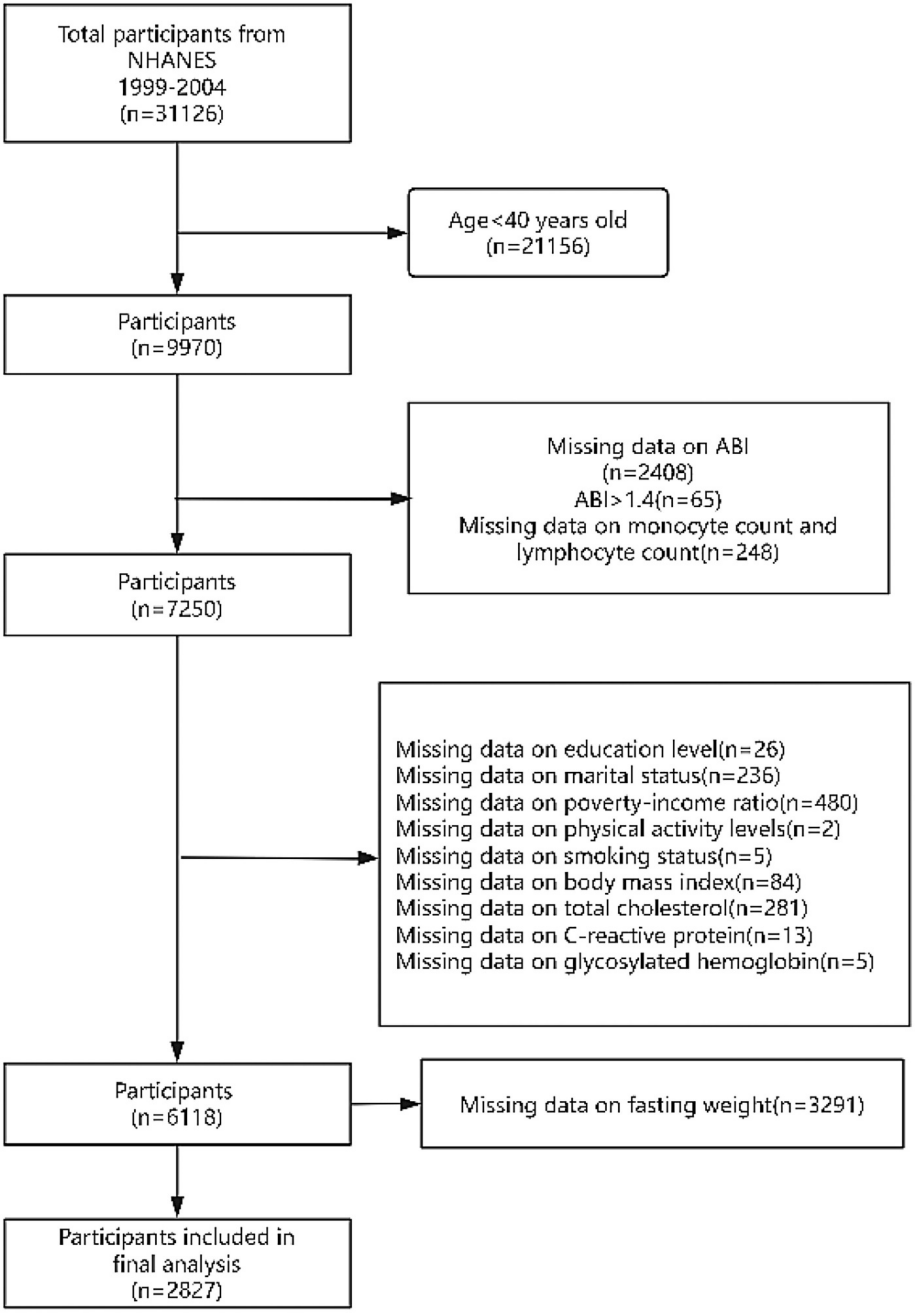
The flow chart of the study.

In total, 31,126 individuals participated in the in-home interviews. Finally, 2,827 individuals were included in the study (Figure 1). The study included eligible individuals aged 40 years or older and had a prevalence rate of PAD at 3.96%. Table 1 summarizes the weighted baseline characteristics of 2,827 participants stratified by tertiles of the MLR. Participants in the highest MLR tertile (T3) were significantly older, predominantly male, and exhibited elevated cardiovascular risk profiles compared to those in T1 (all *p* < 0.001). Notably, the prevalence of PAD increased across MLR tertiles, with T3 showing the highest rate (5.87% vs. 2.21% in T1, *p* < 0.001). Significant differences were also observed in race, inflammatory markers, smoking status, and comorbidities, whereas distributions of education level, marital status, income, and physical activity were comparable across groups. These findings suggest that higher MLR levels are associated with older age, male sex, and a more adverse cardiometabolic phenotype.

**Table 1:** Weighted baseline characteristics by the monocyte-lymphocyte ratio (MLR) tertiles (T).

Variables	Total	MLR(T1)	MLR(T2)	MLR(T3)	*p*-Value
	(*n* = 2,827)	(*n* = 844)	(*n* = 967)	(*n* = 1,016)	
Gender					<0.001
Male	1,445 (48.67)	313 (34.51)	504 (49.21)	628 (59.35)	
Female	1,382 (51.33)	531 (65.49)	463 (50.79)	388 (40.65)	
Age (years)	55.83 (0.31)	53.69 (0.42)	55.26 (0.43)	58.06 (0.50)	<0.001
Race					<0.001
Mexican American	597 (4.47)	231 (6.46)	224 (4.77)	142 (2.61)	
Other Hispanic	103 (4.22)	36 (5.56)	34 (3.11)	33 (4.23)	
Non-Hispanic white	1,604 (78.48)	354 (67.34)	560 (82.57)	690 (83.34)	
Non-Hispanic black	438 (8.54)	182 (12.77)	131 (7.31)	125 (6.37)	
Other races	85 (4.29)	41 (7.88)	18 (2.24)	26 (3.44)	
Education level					0.590
Less than high school	489 (7.33)	165 (8.42)	169 (6.89)	155 (6.90)	
High school diploma or GED	1,047 (37.66)	313 (36.31)	354 (37.08)	380 (39.30)	
More than high school	1,291 (55.01)	366 (55.28)	444 (56.03)	481 (53.80)	
MS					0.470
Married/Living with partner	1,926 (72.91)	566 (70.94)	674 (74.86)	686 (72.57)	
Widowed/Divorced/Separated	758 (22.34)	239 (24.58)	241 (20.11)	278 (22.72)	
Never married	143 (4.76)	39 (4.48)	52 (5.03)	52 (4.71)	
PIR					0.20
Low income	673 (16.04)	216 (17.36)	226 (14.54)	231 (16.44)	
Medium income	1,086 (34.05)	324 (34.11)	366 (32.52)	396 (35.48)	
High income	1,068 (49.91)	304 (48.53)	375 (52.94)	389 (48.07)	
Physical activity					0.044
Sedentary	1,269 (37.40)	407 (41.21)	414 (34.19)	448 (37.50)	
Moderate	892 (33.65)	243 (29.58)	296 (33.82)	353 (36.70)	
Vigorous	666 (28.95)	194 (29.21)	257 (31.99)	215 (25.80)	
BMI, kg/m^2^	28.36 (0.17)	28.62 (0.31)	28.60 (0.22)	27.93 (0.24)	0.110
Total cholesterol, mg/dL	209.27 (1.23)	215.74 (2.12)	209.36 (1.40)	204.04 (1.62)	<0.001
HbA1c	5.60 (0.03)	5.65 (0.04)	5.60 (0.04)	5.55 (0.03)	0.442
Hypertension					<0.213
No	1,822 (67.33)	568 (68.28)	641 (69.28)	613 (64.70)	
Yes	1,005 (32.67)	276 (31.72)	326 (30.72)	403 (35.30)	
Diabetes					0.190
No	2,534 (90.52)	748 (89.23)	869 (92.09)	917 (90.02)	
Yes	293 (9.48)	96 (10.77)	98 (7.91)	99 (9.98)	
Smoking					0.046
Never	1,287 (45.21)	418 (46.57)	438 (45.71)	431 (43.63)	
Former	987 (33.43)	240 (28.65)	349 (33.37)	398 (37.26)	
Current	553 (21.37)	186 (24.78)	180 (20.92)	187 (19.11)	
CVD					0.007
No	2,424 (88.20)	768 (91.76)	826 (87.92%)	830 (85.66)	
Yes	403 (11.80)	76 (8.24)	141 (12.08)	186 (14.34)	
Monocyte count	0.54 (0.01)	0.44 (0.01)	0.53 (0.01)	0.64 (0.01)	<0.001
Lymphocyte count	1.80 (1.50, 2.20)	2.30 (1.80, 2.70)	1.80 (1.60, 2.20)	1.50 (1.20, 1.80)	<0.001
CRP	0.23 (0.10, 0.49)	0.22 (0.09, 0.52)	0.22 (0.10, 0.46)	0.24 (0.11, 0.51)	0.311
MLR	0.31 (0.00)	0.19 (0.00)	0.28 (0.00)	0.44 (0.01)	<0.001
PAD					<0.001
No	2,663 (96.04)	814 (97.79)	917 (96.58)	932 (94.13)	
Yes	164 (3.96)	30 (2.21)	50 (3.42)	84 (5.87)	

Data are presented as *n* (%), median (IQR), or mean (mean.std.error).Data are presented as unweighted number (weighted percentage) for categorical variables and mean (standard error) for continuous variables.

NHANES, National Health and Nutrition Examination Survey; MS, marital status; PIR, poverty income ratio; BMI, body mass index; CVD, cardiovascular disease; CRP, C-reactive protein; GED, general educational development; HbA1c, glycosylated hemoglobin; PAD, peripheral arterial disease.

### 3.2 Association between MLR and PAD

The univariate analysis revealed significant associations between various factors, including age, marital status, smoking habits, family income, physical activity level, education level, total cholesterol levels, CRP levels, monocyte count, HbA1c levels, CVD presence, hypertension, and diabetes with the occurrence of PAD (Table 2). Incorporating NHANES sampling weights, we evaluated the association between MLR and PAD across five adjustment models. In the weighted analysis, MLR as a continuous variable showed a strong association with PAD in unadjusted and partially adjusted models (e.g., Model 4: OR = 4.00, *p* = 0.006), but this relationship was attenuated and became non-significant after full adjustment including age (Model 5: OR = 1.99, *p* = 0.143). When analyzed categorically, participants in the highest MLR tertile (T3) maintained a significantly elevated odds of PAD even in the fully adjusted model (Model 5: OR = 2.25, *p* = 0.008), with a significant trend across tertiles (*p* = 0.008). These weighted results indicate that while elevated MLR is associated with PAD, its effect is substantially confounded by age and other clinical factors (Table 3).

**Table 2:** Association of covariates and PAD risk.

Variable	OR-95% CI	*P-value*
Gender		
Male	1 (reference)	
Female	1.015 (0.821–1.254)	0.8907
Age (years), Mean ± SD	1.082 (1.072–1.093)	<0.001
Race		
Mexican American	1 (reference)	
Other Hispanic	0.805 (0.394–1.646)	0.5528
Non-Hispanic white	1.297 (0.966–1.742)	0.0842
Non-Hispanic black	1.798 (1.278–2.529)	8 × 10^−4^
Other races	0.592 (0.234–1.495)	0.2676
Education level		
Less than high school	1 (reference)	
High school diploma or GED	0.698 (0.535–0.911)	0.008
More than high school	0.437 (0.33–0.579)	<0.001
MS		
Married/Living with partner	1 (reference)	
Widowed/Divorced/Separated	2.009 (1.617–2.497)	<0.001
Never married	0.589 (0.309–1.122)	0.1073
PIR		
Low income	1 (reference)	
Medium income	0.808 (0.633–1.03)	0.0854
High income	0.41 (0.308–0.546)	<0.001
Physical activity		
Sedentary	1 (reference)	
Moderate	0.6 (0.471–0.764)	<0.001
Vigorous	0.278 (0.193–0.399)	<0.001
BMI, kg/m^2^, Mean ± SD	0.987 (0.968–1.007)	0.2031
Total cholesterol, mg/dL, Mean ± SD	0.998 (0.995–1.001)	0.1232
CRP, Median (IQR)	1.233 (1.13–1.346)	<0.001
HbA1c, %	1.222 (1.142–1.308)	<0.001
Hypertension		
No	1 (reference)	
Yes	2.843 (2.291–3.529)	<0.001
Diabetes		
No	1 (reference)	
Yes	2.509 (1.957–3.217)	<0.001
Smoking		
Never	1 (reference)	
Former	1.893 (1.481–2.42)	<0.001
Current	1.907 (1.435–2.535)	<0.001
CVD		
No	1 (reference)	
Yes	3.14 (2.497–3.948)	<0.001
Lymphocyte count, Median (IQR)	0.996 (0.928–1.068)	0.9006
Monocyte count, Mean ± SD	3.273 (2.141–5.003)	<0.001

CI, confidence interval.

**Table 3:** Weighted association between MLR and PAD.

Variable	MLR	*P-value*	T1 (0.04–0.23)	T2 (0.24–0.32)	*P-value*	T3 (0.33–2.20)	*P-value*	Trend.test
								*P-value*
	OR (95% CI)		OR (95% CI)	OR (95% CI)		OR (95% CI)		
Model 1	6.52 (2.60–16.33)	<0.001	1(Ref)	1.57 (0.91–2.70)	0.105	2.76 (1.60–4.75)	<0.001	<0.001
Model 2	6.58 (2.64–16.42)	<0.001	1(Ref)	1.73 (0.99–3.02)	0.054	2.95 (1.68–5.19)	<0.001	<0.001
Model 3	5.62 (2.30–13.71)	<0.001	1(Ref)	1.77 (1.00–3.13)	0.050	2.95 (1.68–5.19)	<0.001	<0.001
Model 4	4.00 (1.57–10.22)	0.006	1(Ref)	1.77 (0.98–3.20)	0.057	2.81 (1.58–4.99)	0.001	0.001
Model 5	1.99 (0.78–5.07)	0.143	1(Ref)	1.64 (0.90–2.98)	0.101	2.25 (1.27–3.99)	0.008	0.008

T, tertiles; OR, odds ratio; CI, confidence interval; Ref, reference.

Model 1 adjusted for none.

Model 2 adjusted for gender + race + education level + MS + PIR + physical activity.

Model 3 adjusted for Model 2 + BMI + total cholesterol + CRP + HbA1c + smoking status.

Model 4 adjusted for Model 3 + cardiovascular disease + hypertension + diabetes.

Model 5 adjusted for Model 4 + age.

**Figure 2:**
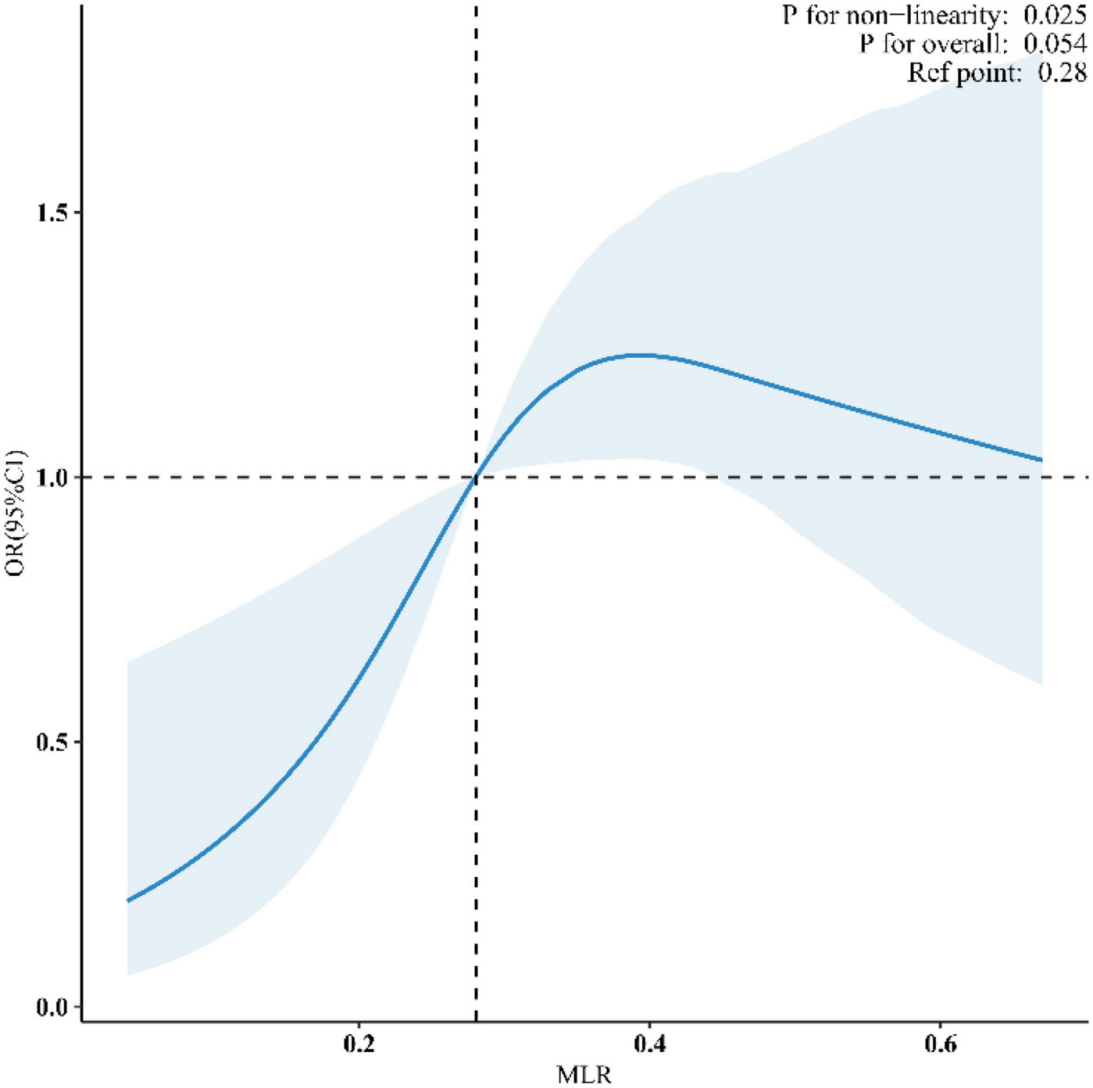
Nonlinear association of MLR with PAD prevalence (weighted analysis). The fitted curve and shaded band indicate the estimated odds ratio and 95% confidence intervals, respectively. Covariates adjusted in the model include demographic factors (age, sex, race, education, marital status, income), lifestyle factors (physical activity, smoking), anthropometric and biochemical measures (BMI, total cholesterol, CRP, HbA1c), and clinical comorbidities (hypertension, diabetes, CVD). The reference MLR value was set at 0.28. The association demonstrates a nonlinear pattern (*P* for non-linearity = 0.025). The shaded confidence band widens at the lower and upper extremes of MLR due to fewer data points, indicating greater uncertainty in the estimated relationship in these regions.

Figure 2 presents the weighted restricted cubic spline analysis evaluating the dose-response relationship between the MLR and PAD prevalence. After adjusting for demographic, lifestyle, anthropometric, biochemical, and clinical covariates, the analysis revealed a nonlinear association (*P* for non-linearity = 0.025). The odds ratio (OR) for PAD showed a gradual increase with rising MLR values, particularly above the reference point of 0.28. However, the overall association did not reach statistical significance (*P* for overall = 0.054). These results suggest a complex, non-linear relationship between MLR and PAD risk in this nationally representative sample, with the inflammatory marker exhibiting a threshold effect rather than a simple linear trend. It is important to note that the confidence intervals widen substantially at both the lower and upper extremes of the MLR distribution, reflecting fewer observations and greater uncertainty in these ranges. Consequently, the precise shape of the curve at the tails should be interpreted with caution.

Table 4 presents the threshold analysis of MLR in relation to PAD based on weighted restricted cubic spline modeling. Using an MLR cut-point of 0.343 derived from the RCS curve, the analysis demonstrated a significant association with PAD at lower MLR levels (MLR < 0.343: OR = 1.62, 95% CI 1.24–2.11, *p* < 0.001). In contrast, no statistically significant association was observed at higher MLR values (MLR ≥ 0.343: OR = 0.94, 95% CI 0.64–1.38, *p* = 0.738). The log-likelihood ratio test confirmed the presence of a significant threshold effect (*p* = 0.009). These findings suggest that the association between MLR and PAD follows a nonlinear pattern, with increased risk primarily observed below the threshold of 0.343.

**Table 4:** Threshold analysis of MLR and PAD based on weighted RCS.

MLR	Adjust Model	
	OR (95% CI)	*P-value*
<0.343	1.62 (1.24–2.11)	<0.001
≥0.343	0.94 (0.64–1.38)	0.738
Log-likelihood ratio test		0.009

OR, odds ratio; CI, confidence interval. Covariates adjusted in the model include all demographic factors, lifestyle factors, anthropometric and biochemical measures, and clinical comorbidities. Only 99% of the data is displayed.

### 3.3 Stratification analysis

**Figure 3:**
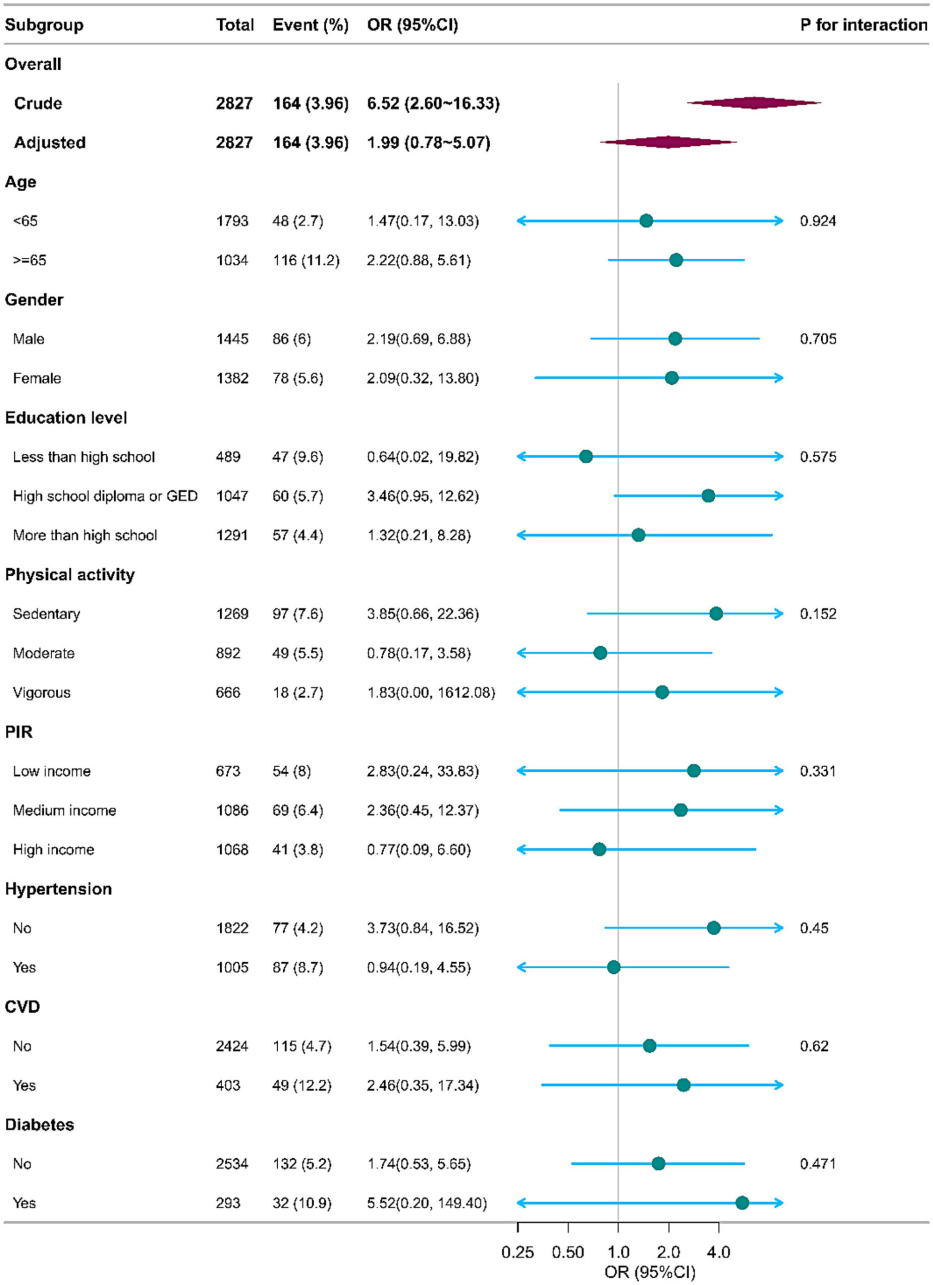
Forest plot of the weighted subgroup analysis for the association between MLR and PAD.

Figure 3 presents weighted subgroup analyses evaluating the MLR–PAD association across demographic and clinical variables. The fully adjusted OR for the overall cohort was 1.99 (95% CI: 0.78–5.07). Subgroup-specific estimates remained generally consistent, with no statistically significant variation observed by age, sex, education, physical activity, income, hypertension, cardiovascular disease, or diabetes status (all *P*-interaction > 0.05). These findings suggest that the relationship between MLR and PAD—while attenuated after full adjustment—does not differ substantially across key population subgroups in this nationally representative sample.

## 4 Discussion

This study examined the relationship between the MLR and the prevalence of PAD in a large, nationally representative sample of U.S. adults. The diminishing strength of this association across various adjustment models—especially the loss of significance after adjusting for age—highlights the intricate interplay between systemic inflammation, aging, and vascular pathology. These findings contribute to the expanding evidence that links immune dysregulation to atherosclerosis and its clinical manifestations.

The robust unadjusted association observed between MLR and PAD (OR =  6.52 in Model 1) aligns with previous studies that identify systemic inflammation as a fundamental aspect of PAD pathogenesis (37). Monocytes play a crucial role in vascular inflammation by promoting endothelial dysfunction through cytokine release and foam cell formation, whereas lymphocytes are essential for regulating plaque stability (38). Consequently, an elevated MLR may indicate a pro-inflammatory state that exacerbates arterial damage.The substantial attenuation of the MLR-PAD association upon adjustment for age underscores that chronological age is a paramount confounder. This observation aligns with the concept of “inflammaging,” where age-associated immune dysregulation—characterized by heightened monocyte activity and reduced lymphocyte diversity—promotes a pro-inflammatory state that accelerates atherosclerosis. Thus, age may represent both a confounding variable and the temporal substrate for the biological process linking immune aging to PAD. Furthermore, our weighted restricted cubic spline analysis revealed a nonlinear relationship, identifying a significant threshold at MLR = 0.343. This suggests that the inflammatory risk reflected by MLR may not increase linearly but rather exhibits a plateau effect beyond a certain level, which could partly explain the attenuation observed in continuous models. The persistent elevation in PAD odds within the highest MLR tertile even after full adjustment underscores that individuals with markedly elevated inflammatory ratios warrant particular clinical attention.This phenomenon, referred to as “inflammaging,” is characterized by chronic low-grade inflammation driven by aging immune cells and oxidative stress (39). For example, aging is associated with heightened monocyte activation and diminished lymphocyte diversity, factors that may increase MLR independently of PAD (40). Our findings corroborate longitudinal studies indicating that age intensifies the associations between inflammatory biomarkers and disease (41).

The tertile-based analysis revealed a clear gradient in PAD risk associated with MLR, with the highest tertile (T3) consistently showing elevated odds (OR = 1.2–1.9). This dose-response pattern is consistent with findings in coronary artery disease (CAD), where the MLR predicted adverse outcomes in adults (42). However, in contrast to CAD, adjustments for metabolic factors and comorbidities only partially attenuated the MLR-PAD association (Models 3–4). Residual confounding from unmeasured variables—such as dietary habits, epigenetic factors, or subclinical infections—may explain this persistent association (43). For instance, metabolites derived from gut microbiota, like trimethylamine N-oxide (TMAO), can modulate monocyte activation and are independently linked to PAD (44), although these factors were not assessed in the present study. Despite these limitations, the MLR's cost-effectiveness and routine availability make it a practical tool for PAD risk assessment, especially in younger populations where age-related confounding is less pronounced. Recent guidelines recommend incorporating inflammatory biomarkers into cardiovascular risk assessments (45), and MLR could complement existing measures such as the ABI. Moreover, emerging mechanistic studies suggest that MLR may reflect specific monocyte subsets involved in plaque rupture (46, 47). Targeting these subsets through immunomodulatory therapies has shown promise in reducing atherosclerotic events (48), raising the possibility that MLR-guided interventions could slow the progression of PAD. Our findings, particularly the threshold effect and the sustained risk in the highest tertile, suggest that MLR may be most informative as a categorical or risk-stratifying marker rather than a continuous linear predictor. In clinical practice, an MLR value exceeding 0.34 or placement in the top population tertile could serve as a pragmatic, low-cost flag for heightened inflammatory burden and associated PAD risk, prompting more thorough vascular assessment or intensification of risk factor management. This persistent association at the extreme of the MLR distribution suggests that while the population-level, linear relationship is heavily confounded by age, a severely elevated MLR remains an informative marker of pathological inflammatory burden, potentially capturing aspects of “inflammaging” not fully accounted for by chronological age alone.

This study provides a detailed, population-level assessment of the MLR in relation to PAD. Although MLR has been examined in other cardiovascular settings, evidence specifically linking it to PAD remains limited. Our work strengthens this evidence base through a methodologically robust approach that incorporates complex survey weighting for national representativeness, sequential multivariable modeling to account for confounding—with particular attention to the role of age—and flexible spline-based analyses to detect non-linear patterns. These analyses not only clarify the magnitude of the association but also reveal a previously undefined inflection point at an MLR of 0.343, offering a more refined perspective on how this accessible inflammatory marker behaves across the spectrum of PAD risk in a representative U.S. cohort. This study has several limitations. First, the cross-sectional design prevents causal inference, and residual confounding may persist. Second, MLR is subject to temporal variability due to acute conditions such as infection or stress, which may lead to underestimation of its true association with PAD (49). Third, while major confounders were adjusted for, unmeasured variables like genetic predisposition could still influence the results (50). Fourth, while we employed sampling weights to enhance national representativeness, residual selection bias from missing data, particularly fasting weight, cannot be entirely ruled out. Finally, the absence of detailed clinical symptom data (e.g., claudication, rest pain) precluded analysis of the relationship between MLR and functional PAD severity. Despite these limitations, the observed association between MLR and PAD suggests its potential as an inexpensive vascular risk marker. Future longitudinal studies should validate its ability to predict both incident PAD and adverse clinical outcomes (e.g., critical limb ischemia, amputation) to establish its clinical utility.

Furthermore, while this study focused on MLR, other inflammatory biomarkers such as NLR and PLR have also been implicated in cardiovascular diseases. Future studies would benefit from directly comparing the predictive values of MLR, NLR, and PLR for PAD, and investigating their potential interactions and collinearity, to identify the most informative inflammatory signature for risk stratification.

## 5 Conclusions

Our nationally representative analysis reveals that the association between MLR and PAD is substantially confounded by age, which likely represents both a confounder and a marker of underlying “inflammaging”. While isolating a purely independent effect of MLR is therefore challenging, a markedly elevated MLR (particularly in the highest population tertile) robustly identifies individuals at significantly increased PAD risk. This supports its potential utility as a simple clinical indicator of immune-vascular dysregulation. Future longitudinal studies should validate the identified threshold and assess MLR's prognostic value for clinical PAD endpoints.

## Data Availability

Publicly available datasets were analyzed in this study. This data can be found here: http://www.cdc.gov/nchs/nhanes/.
