# Supplementary material for: Association of monocyte-lymphocyte ratio with peripheral arterial disease in US participants: a retrospective cross-sectional study
Source: Front Cardiovasc Med. 2026 Jan 8;12:1613138. doi: 10.3389/fcvm.2025.1613138 (PMC12823977; doi:10.3389/fcvm.2025.1613138)
Supplement: Supplementary file 1 [file Table1.docx]

Supplementary Table 1. Summary of Current Evidence on the Role of MLR in Cardiovascular Diseases

| Cardiovascular Disease | Study Design | Population | Key Findings | Reference |
| --- | --- | --- | --- | --- |
| **Coronary Artery Disease (CAD)** | Prospective observational study | consecutive patients from a single tertiary care hospital | MLR was significant predictors of severe CAD in univariate analysis | Bani Hani DA, et al. Lymphocyte-based inflammatory markers: Novel predictors of significant coronary artery disease. Heart Lung. 2024. |
| **CAD Prognosis** | Systematic review and meta-analysis | Patients with coronary heart disease (CHD) from 19 included studies | Higher MLR significantly associated with increased risk of major adverse cardiovascular events (MACE) in CHD patients | Vakhshoori M, et al. Prognostic impact of monocyte-to-lymphocyte ratio in coronary heart disease: a systematic review and meta-analysis. J Int Med Res. 2023;51(10):3000605231204469. |
| **Heart Failure** | Prospective cohort study | 334,674 individuals from UK Biobank | MLR positively associated with risk of atrial fibrillation and heart failure | Luo Y, et al. The association between blood count based inflammatory markers and the risk of atrial fibrillation, heart failure and cardiovascular mortality. Sci Rep. 2024;14:40128300. |
| **Ischemic Stroke** | Retrospective cohort study | 395 participants with ischemic stroke | MLR was significantly correlated with severity of carotid stenosis in ischemic stroke patients | Zuo B, et al. Monocyte/lymphocyte ratio is associated with carotid stenosis in ischemic stroke: A retrospective analysis. Brain Behav. 2019;9(11):e01429. |
| **Peripheral Arterial Disease (PAD)** | Cross-sectional | NHANES participants | MLR associated with PAD prevalence, but association attenuated after full adjustment for covariates | Current Study |
