# Supplementary material for: Association of monocyte-lymphocyte ratio with peripheral arterial disease in US participants: a retrospective cross-sectional study
Source: Front Cardiovasc Med. 2026 Jan 8;12:1613138. doi: 10.3389/fcvm.2025.1613138 (PMC12823977; doi:10.3389/fcvm.2025.1613138)
Supplement: Supplementary file 2 [file Table2.docx]

Supplementary Table 2 Assessment of multicollinearity and covariate influence in logistic regression models for PAD.

| **Term1** | **Coeff1** | **Chang.percentage1** | **Term2** | **Coeff2** | **Chang.percentage2** | **VIF** |
| --- | --- | --- | --- | --- | --- | --- |
| **Crude** | 1.7 | Ref. | **Full** | 0.46 | Ref. | 1.187 |
| **Gender** | 1.72 | 1 | **Gender** | 0.37 | -20.6 | 1.342 |
| **Age** | 0.47 | -72.5 | **Age** | 1.19 | 157.8 | 1.6 |
| **Race** | 1.74 | 2.5 | **Race** | 0.39 | -15.9 | 1.726 |
| **BMI** | 1.63 | -4.1 | **BMI** | 0.49 | 5.5 | 1.197 |
| **Total cholesterol** | 1.63 | -4.3 | **Total cholesterol** | 0.47 | 1 | 1.126 |
| **CRP** | 1.59 | -6.5 | **CRP** | 0.58 | 24.8 | 1.062 |
| **HbA1c** | 1.76 | 3.3 | **HbA1c** | 0.47 | 2.6 | 1.427 |
| **Education level** | 1.82 | 7.2 | **Education level** | 0.38 | -17 | 1.644 |
| **MS** | 1.69 | -0.5 | **MS** | 0.46 | -0.9 | 1.422 |
| **PIR** | 1.73 | 1.9 | **PIR** | 0.47 | 1.7 | 1.422 |
| **Hypertension** | 1.55 | -8.8 | **Hypertension** | 0.5 | 8.8 | 1.142 |
| **Diabetes** | 1.69 | -0.4 | **Diabetes** | 0.47 | 2.3 | 1.408 |
| **Physical activity** | 1.69 | -0.5 | **Physical activity** | 0.43 | -6.8 | 1.136 |
| **Smoking** | 1.59 | -6.4 | **Smoking** | 0.36 | -22.2 | 1.46 |
| **CVD** | 1.41 | -17.1 | **CVD** | 0.54 | 17.8 | 1.145 |
